# Effect of Indigenous Non-*Saccharomyces* Yeasts on Lipid Compositions of Maraština Wine

**DOI:** 10.3390/foods14020269

**Published:** 2025-01-15

**Authors:** Ana Boban, Urska Vrhovsek, Domenico Masuero, Vesna Milanović, Irena Budić-Leto

**Affiliations:** 1 Institute for Adriatic Crops and Karst Reclamation, 21000 Split, Croatia; aboban@krs.hr; 2 Metabolomics Unit, Research and Innovation Centre, Fondazione Edmund Mach, 38010 San Michele all’Adige, Italy; urska.vrhovsek@fmach.it (U.V.); domenico.masuero@fmach.it (D.M.); 3 Department of Agricultural, Food and Environmental Sciences, Polytechnic University of Marche, 60131 Ancona, Italy; v.milanovic@univpm.it

**Keywords:** isolates, non-*Saccharomyces* yeasts, lipids, UHPLC-MS/MS, monoculture, sequential fermentations

## Abstract

This study is the first to investigate the impact of indigenous non-Saccharomyces yeasts, including *Hypopichia pseudoburtonii*, *Metschnikowia sinensis*/*shanxiensis*, *Metschnikowia chrysoperlae*, *Metschnikowia pulcherrima*, *Lachancea thermotolerans*, *Hanseniaspora uvarum*, *Hanseniaspora guilliermondii*, *Hanseniaspora pseudoguilliermondii*, *Pichia kluyveri*, and *Starmerella apicola* on the lipid composition of sterile Maraština grape juice and wines using the UHPLC-MS/MS method. Yeasts were tested in monoculture and sequential fermentations alongside commercial *Saccharomyces cerevisiae*. Indigenous non-Saccharomyces yeasts showed the potential to improve fermentation performance and enable the development of new wine styles through the biosynthesis of an unsaturated fatty acid pathway, which was identified as the most significant pathway. In monoculture fermentations, *L. thermotolerans*, *H. uvarum*, *H. guilliermondii*, *H. pseudoguilliermondii*, and *P. kluyveri* significantly reduced lignoceric acid, potentially influencing wine aroma through the formation of esters and higher alcohols. *Hyp. pseudoburtonii*, *M. chrysoperlae*, *M. pulcherrima*, *P. kluyveri*, and *S. apicola* increased the demand for lipids, such as stearic acid, which may help preserve membrane permeability by integrating into the membrane in response to ethanol shock. The most significant impact on free fatty esters was observed in fermentations with *H. pseudoguilliermondii*. Furthermore, *L. thermotolerans* in sequential fermentations significantly reduced arachidic, stearic, and palmitic acid. *P. kluyveri* reduced the content of erucic and linoleic acid.

## 1. Introduction

Nowadays, non-*Saccharomyces* yeasts are widely used yeasts in the winemaking industry. Many species possess valuable enzymatic activity, antimicrobial properties, and can enhance aroma complexity and improve the sensory properties of wines [1,2]. Among chemical compounds, lipids have been identified as limiting factors and essential nutrients for increasing the viability of yeasts [3]. They represent an important group of molecules that directly contribute to the development of wine aroma, and recent studies suggest they may also play a role in wine mouthfeel [4].

Lipids are hydrophobic molecules found in microorganisms, plants, and animals. They can be classified into eight categories based on their chemical building blocks: fatty acyls, glycerolipids, glycerophospholipids, sphingolipids, saccharolipids, polyketides, sterols, and prenol lipids [5]. These molecules play crucial roles as an energy source, structural components, regulators of biological processes, participants in signaling, and facilitate cellular communication [6,7]. In wine, lipids are primarily derived from grape tissues and yeast cell walls [8]. The lipid content sourced from grape tissues accounts for 0.15% to 0.24% of the fresh weight [5]. Additionally, lipids are essential components of yeast cell membranes, with concentrations ranging from less than 5% to over 15% of dry weight, depending on the species [9]. Pérez-Navarro et al. [10] reported that lipid composition diversity in grapes depends on grape tissue type and cultivars, regardless of berry color. Additionally, various lipids belonging to free fatty acids, glycerolipids, glycerophospholipids, and triterpenoids were identified. Arita et al. [11] discovered a strong correlation between the heat requirement of grapevine cultivars and the degree of fatty acid desaturation. Lipids, along with membrane proteins, form the structural framework of biological membranes [12]. The lipid composition of the plasma membrane in *Saccharomyces cerevisiae* has been extensively studied [13], as well as in *Pichia pastoris* [14] and *Candida albicans* [15]. The most abundant lipids in yeast are phospholipids, sterols, and sphingolipids [16]. Standard winemaking practices influence lipid metabolism and lipid concentrations. Furthermore, the literature mainly explores insight into the impact of fermentation temperatures [17,18] and the addition of yeast products (yeast cell walls, inactivated yeasts, and yeast hulls) and lipids supplementation (phytosterols and fatty acids), on wine lipid composition [19,20,21]. Increasing the fermentation temperature can significantly raise total lipid content [18], while lipid supplementation can enhance fermentation performance and the metabolomic footprint in wine [21]. Lipids are the primary source of nutrition for yeast during fermentation and contribute to the formation of volatile aroma compounds during fermentation [22,23]. Fatty acid peroxidation produces aldehydes, ketones, and alcohols, with C6 compounds (such as aldehydes and alcohols) imparting green, vegetal, and herbaceous notes to both red and white wines. Additionally, lipids are precursors to esters, which create fruity aromas, and lactones, which add coconut-like notes [22,23]. Besides the contribution to wine aroma, lipids help modulate astringency by interacting with tannins in wine. These interactions can decrease the perception of astringency, contributing to a smoother mouthfeel [24]. Additionally, many analytical techniques for lipid determination have been developed in the past [25,26], though they often require lengthy sample preparation or compound derivatization. In a previous study [27], we reported the lipid composition of Maraština berry skin (*Vitis vinifera* L.), an autochthonous Croatian variety, from 11 vineyards located within the Maraština cultivation area, representing the first insight into this variety’s lipid profile. Due to the chemical complexity of the wine matrix and the diversity of lipid compounds, ultra-high-performance liquid chromatography (UHPLC) coupled with mass spectrometry (MS) has become the most efficient method for lipidomic analysis [10]. The UHPLC-MS/MS method demonstrated excellent sensitivity, specificity, and an extensive dynamic range [28]. The Maraština variety is regarded as the second most important grape variety in the Dalmatia wine region, following Pošip, due to its significant potential for producing high-quality wines. It is capable of producing both high-quality monovarietal and dessert wines. Commercial wines made from Maraština are known for their distinct characteristics, including a more intense yellow color, fuller body, higher viscosity, and increased astringency compared to other white varieties such as Istrian Malvasia, Chardonnay, and Muscat Blanca [29]. While Maraština does not exhibit as pronounced aromatic potential as some other varieties, a study by Budić-Leto et al. [30] has highlighted that Maraština grapes contain elevated levels of aromatic compounds, particularly terpenic compounds and norisoprenoids, which are primarily present in their glycosidic form. This suggests that Maraština possesses a significant aromatic potential.

Considering the literature data, the studies were based on monitoring how different lipid supplements, yeast extract, fermentation temperatures, and oxygen exposure affect lipids and wine characteristics. However, a knowledge gap exists regarding how lipid composition in grape juice and Maraština wines is affected by the inoculation of different indigenous non-*Saccharomyces* yeasts and various fermentation practices. To address this, this study investigates the impact of indigenous non-*Saccharomyces* yeasts isolated from Maraština grapes on the lipidomic profile of wines using a targeted UHPLC-MS/MS approach. The tested species were *Metschnikowia pulcherrima*, *Lachancea thermotolerans*, *Hanseniaspora uvarum*, *Hanseniaspora guilliermondii*, *Hanseniaspora pseudoguilliermondii*, *Pichia kluyveri*, and *Starmerella apicola*, as well as three species not previously studied in oenological environments such as *Hypopichia pseudoburtonii*, *Metschnikowia chrysoperlae*, and *Metschnikowia sinensis/shanxiensis.* Both monoculture and sequential fermentation practices were employed, using sterile Maraština grape juice as the fermentation substrate. Sterile Maraština grape juice was used as a model solution to elucidate the behavior of certain yeast species and their impact on lipid content. Fermentations with commercial yeasts *S. cerevisiae* EC 1118, *M. pulcherrima* Flavia, and *L. thermotolerans* Octave were used as control treatments. Moreover, the study precisely evaluated each yeast species’ performance across different fermentation practices and introduced new insights into how indigenous non-*Saccharomyces* yeasts can contribute to wine aroma through lipid synthesis or degradation.

## 2. Materials and Methods

### 2.1. Chemicals

Chemicals for the preparation of yeast growth media, including bacteriological peptone and yeast extract, were purchased from Biolife Italiana S.r.l. (Milan, Italy), while bacteriological dextrose was obtained from Oxoid (Hampshire, UK). Standards for UHPLC analysis were purchased from Sigma-Aldrich (Sigma-Aldrich, Milan, Italy). Acetonitrile (ACN, LC–grade), 2-propanol (IPA), methanol (CH_3_OH, LC-MS grade), and chloroform (CHCl_3_) were also purchased from Sigma-Aldrich. Formic acid (HCOOH) and ammonium formate (NH_4_COOH) additives for LC-MS were also from FLUKA Sigma-Aldrich. All aqueous solutions, including the UHPLC mobile phase, were prepared with water purified using a Milli-Q system (Millipore, Vimodrone, Milan, Italy).

### 2.2. Non-Saccharomyces Isolates

This study includes ten indigenous non-*Saccharomyces* yeasts from the collection established in 2021 at the Institute for Adriatic Crops and Karst Reclamation (Split, Croatia). The yeasts were originally isolated from Maraština grapes and identified at the molecular level through sequencing of the ITS1-5.8S-ITS2 rDNA region, following the methodology outlined by Milanović et al. [31]. The isolates used were *Hyp. pseudoburtonii* N-11, *M. chrysoperlae* K-11, *M. sinensis/shanxiensis* P-7, *M. pulcherrima* K-6, *L. thermotolerans* P-25, *H. uvarum* Z-7, *H. guilliermondii* N-29, *H. pseudoguilliermondii* V-13, *P. kluyveri* Z-3, and *S. apicola* VP-8.

The indigenous yeasts, retrieved from glycerol stocks preserved at −80 °C, were inoculated into YPD broth (10 g/L yeast extract, 20 g/L peptone, 20 g/L dextrose) as previously described by Boban et al. [32]. In brief, the isolates underwent two rounds of preculturing at 25 °C. Biomass was collected by centrifugation (Hettich^®^ Universal 320/320R centrifuge, Andreas Hettich GmbH & Co., Tuttlingen, Germany) at 1520× *g* at 4 °C for 5 min. Subsequently, the cell pellet was separated from the supernatant and resuspended in sterile peptone water. The yeast concentrations were measured using a Varian Cary^®^ 50 UV-Vis Spectrophotometer (Agilent Technologies Inc., Santa Clara, CA, USA) at wavelengths of 600 nm. Commercial yeasts, *Saccharomyces cerevisiae* EC 1118 (*Sc*), *M. pulcherrima* Flavia (*Mp* Flavia), and *L. thermotolerans* Octave (*Lt* Octave) (Lallemand Inc., Montreal, QC, Canada), were rehydrated following the manufacturer’s protocols.

### 2.3. Experimental Design

The grapes from the autochthonous Croatian white variety Maraština (*Vitis vinifera* L.), were harvested in September 2022 from a vineyard in the North Dalmatia winegrowing subregion (43°52′49″ N 15°55′29″ E). The grapes were harvested at technological maturity, with a must density of 99 Oe° and concentrations of glucose and fructose equal to 1:1. After being destemmed and crushed, the grapes were treated with potassium metabisulfite to achieve a total sulfur dioxide concentration of approximately 50 mg/L. The grape must was left for 24 h at 4 °C in the winery for cold stabilization. Following cold stabilization, the grape juice was sterilized by sterile filtration using a PALL filler (0.45 µm) at the Institute for Adriatic Crops laboratory. Under sterile conditions, the yeast assimilable nitrogen (YAN) concentration was adjusted to 250 mg/L using (NH_4_)_2_HPO_4_. The sterile grape juice had a total acidity of 4.33 g/L, expressed as tartaric acid, and a pH of 3.35, measured with the Lyza 5000 Wine analyzer (Anton Paar GmbH, Graz, Austria).

Laboratory-scale fermentations were carried out in triplicate using 500 mL of sterile Maraština grape juice in Erlenmayer flasks, which were sealed with sterilized porous caps. Fermentations were performed at 20 °C until completion for each isolate under two fermentation practices: monoculture and sequential fermentations. Monoculture fermentations were conducted for all ten non-*Saccharomyces* isolates. Furthermore, sequential fermentations were conducted with seven isolates that previously demonstrated desirable oenological properties, such as growth at different temperatures, high ethanol and SO_2_ tolerance, low production of hydrogen sulfide and acetic acid, and enhanced enzymatic activity [33]. This selection included *M. chrysoperlae*, *M. sinensis/shanxiensis*, *M. pulcherrima*, *L. thermotolerans*, *H. uvarum*, *H. guilliermondii*, and *P. kluyveri*. For both fermentation practices, the grape juice was inoculated with approximately 10^7^ cells/mL of the respective non-*Saccharomyces* isolate. In sequential fermentations, *S. cerevisiae* was inoculated in ferments at the same concentration when the ethanol level reached 2–3% *v/v* during alcoholic fermentation. Control fermentations were performed using commercial yeast strains. At the end of alcoholic fermentation, defined by a residual sugar concentration of less than 5 g/L, samples were collected using a sterile plastic pipette and transferred into 50 mL Falcon tubes, then stored at −80 °C until UHPLC-MS/MS analysis.

### 2.4. UHPLC-MS/MS Analysis of Lipids

Lipid analysis was conducted using a UHPLC system with a Dionex 3000 chromatograph (Thermo Fisher Scientific, Waltham, MA, USA), coupled with an API 5500 triple-quadrupole mass spectrometer equipped with an ESI source (Sciex, Concord, Vaughan, ON, Canada). The procedure followed the method described by Della Corte et al. [34], with minor modifications. A 0.5 mL aliquot of the wine sample was placed in a 20 mL glass headspace vial, along with 1.5 mL of methanol, and vortexed for 30 s (IKA-Werke GmbH & Co. KG, Staufen, Germany). Subsequently, 3.0 mL of chloroform containing BHT (500 mg/L) as an antioxidant and 1.25 mL of Milli-Q water were added. The solution was spiked with 10 µL of stearic acid d3 (100 µg/mL) as an internal standard (IS). The samples underwent two extraction rounds using an orbital shaker (Grant-bio, Amsterdam, The Netherlands) for 15 min, and the lipid-rich lower layer was collected. The collected samples were centrifuged (Thermo Fisher Scientific, Waltham, MA, USA) for 2 min and evaporated to dryness under nitrogen. The dried samples were reconstituted in 300 µL of a mixture of acetonitrile/2-propanol/Milli-Q water (65:30:5 *v*/*v*/*v*), containing cholesterol d7 (1.0 µg/mL), and filtered through a 0.22 µm membrane into 2 mL HPLC amber vials with a glass insert. Lipid separation was achieved using a 2.7 µm, 150 × 2.1 mm RP Ascentis Express column (Sigma-Aldrich, Milan, Italy) at 55 °C. A 5.0 µL injection volume was used, and the samples were maintained in an autosampler at 10 °C during the analyses. The mobile phase and chromatographic conditions were described by Della Corte et al. [34]. Instrument control and data acquisition were managed using Analyst™ software version 1.6.1. (Applera Corporation, Norwalk, CT, USA), while data processing was carried out using MultiQuant version 3.1 (Sciex, Concord, Vaughan, ON, Canada).

### 2.5. Data Analysis

Statistical data analysis was performed using Software Statistica (StatSoft, Tulsa, OK, USA) v.12.0. Data normality was assessed using the Kolmogorov–Smirnov test. One-way analysis of variance (ANOVA) was applied to evaluate differences in lipid profiles among yeast isolates, commercial yeasts, and the initial grape juice. Post hoc multiple comparisons were carried out using Tukey’s range test to identify specific distinctions, with a significance level set at *p* < 0.05. Multivariate analysis was performed with MetaboAnalyst 5.0 (University of Alberta, Edmonton, AB, Canada) [35].

## 3. Results and Discussion

The concentration of lipid compounds in Maraština grape juice and wines obtained from monoculture and sequential fermentation practices were displayed in Table 1 and Table 2 as follows. Implementing a targeted approach with the UHPLC-MS/MS method, 20 compounds were identified and categorized into different lipid classes, including free fatty acids (11), triterpenoids (1), glycerolipids (1), glycerophospholipids (1), and free fatty esters (6).

### 3.1. Maraština Grape Juice Lipid Content

Grape juice is a water-based, sugar-rich medium containing different classes of metabolites, including lipids. Grapes contain approximately 0.15% to 0.24% lipids, with fatty acids as the dominant lipid subclass [10]. The lipid content of the Maraština grape juice is reported in Table 1 and Table 2. The free fatty acids represented the most abundant lipid subclass, with a concentration of 30.84 mg/L where palmitic acid represented 61% of free fatty acids followed by stearic acid (8.64 mg/L). The lipid content in grape juice can influence acetic acid levels. Deroite et al. [36] reported that adding phytosterols and cell structure components, such as Tween 80, can reduce acetic acid, possibly due to a decrease in acetyl-CoA, the key substrate for lipid metabolism. Comprehensive lipidome profiling of Sauvignon Blanc grape juice reported by Tumanov et al. [37] exhibited that most lipids are present in the form of complex lipids which can be released through the lipolytic activity of microbial enzymes. Furthermore, oleanolic acid, a pentacyclic triterpenoid, accounted for 0.10% of lipid content in grape juice in this study. In contrast, our previous research reported that oleanolic acid was the most abundant lipid compound in Maraština grape skin [27]. This difference can be attributed to the typical white winemaking process for Maraština, which involves no skin contact or seed maceration, as the seeds contain most lipids [38]. Additionally, it is well known that grape variety, viticulture practices, and vintage play crucial roles in determining the final lipid composition of grapes [10,37]. Maraština grape juice contains 0.29 mg/L of 1-linoleoyl-rac-glycerol (1-linoleoyl-rac-GL) as the only glycerolipid. Garrido et al. [39] reported that exposure of the vines to high light increases the concentrations of glycerolipids and glycerophospholipids. Maraština juice contained 0.01 mg/L of 1,2-dioleoyl-sn-glycero-3-phospho-rac-(1-glycerol) sodium salt (1,2-dioleoyl-GLP-Na) which can serve as precursors for the synthesis of sphingolipids and sterols, both essential for yeast growth and viability. Furthermore, fatty acids are mainly present in the free form or esterified as ethyl esters [25] and both forms positively contribute to the sensorial properties of wine directly or as precursors associated with green and fruity aromas [40]. The total concentration of fatty acid esters was 1.49 mg/L, including the methyl oleate (0.44 mg/L), ethyl palmitate (0.44 mg/L), ethyl linoleate (0.04 mg/L), ethyl oleate (0.32 mg/L), and ethyl stearate (0.24 mg/L). Methyl stearate was not detected in grape juice. Initially, lipid components in grape juice play an important role in yeast metabolism, especially after ethanol concentration rises [41]. The total lipid concentration determined by the UHPLC-MS/MS method was 32.63 mg/L, which is four times lower than the concentration reported by Sherman et al. [40] in Pinot Noir and nine times lower in Sauvignon Blanc. However, these values were determined with a different method while Phan et al. [4] lipids are minor constituents with a concentration reported to be less than 0.1% in commercial Pinot noir wines. The different concentrations of lipids in grape juice may modulate yeast metabolism because they can transport amino acids into the yeast cells or ATPase activity [42].

### 3.2. The Role of Yeast Performances on Wine Lipid Content

#### 3.2.1. Monoculture Fermentations

The concentrations of lignoceric, myristic, and stearic acid among the saturated free fatty acids significantly differed from the initial concentrations. All ten supplied indigenous yeasts decrease the concentrations of lignoceric acid. In particular, *L. thermotolerans*, *H. uvarum*, *H. guilliermondii*, *H. pseudoguilliermondii*, and *P. kluyveri* in monoculture fermentations significantly lowered lignoceric acid levels. Furthermore, fatty acids serve as precursors for pleasant wine aroma compounds such as esters and higher alcohols [43]. These results align with our previous study where *H. guilliermondii* and *P. kluyveri* in monoculture produced the highest concentration of total esters, especially isoamyl acetate [32]. Esters like ethyl hexanoate and isoamyl acetate, known for their fruity and floral aromas, may have their formation indirectly impacted by fatty acid precursors like lignoceric acid. In comparison with *S. cerevisiae* control fermentation, fermentation with *P. kluyveri* showed the lowest concentration of myristic acid (0.27 mg/L), which statistically differed from *S. cerevisiae* (0.83 mg/L)*. Hyp. pseudoburtonii*, *M. chrysoperlae*, *M. pulcherrima*, *P. kluyveri*, and *S. apicola* increased stearic acid concentrations compared to the initial concentration (8.64 mg/L). Additionally, stearic acid was utilized most effectively by *H. guilliermondii* (7.87 mg/L), which significantly differed from *S. cerevisiae* (10.87 mg/L). Liu et al. [44] indicated that unsaturated fatty acids can directly impact the synthesis of volatile compounds by regulating the expression of genes, such as ATF1 and EEB1, which are involved in ester biosynthesis. Using UHPLC-MS/MS, five unsaturated acids were identified, including erucic, linolenic, linoleic, oleic, and palmitoleic acids. Statistical differences were observed for erucic (0.03 mg/L) and linoleic acid (0.24 mg/L) in grape juice, where *Hyp. pseudoburtonii* affected erucic acid level (0.02 mg/L) and *L. thermotolerans* affected linoleic acid (0.19 mg/L) and led to their reduction. The utilization of linoleic acid increases amino acid and organic acid biosynthesis [43]. This suggests that these strains may have an increased demand for lipids, such as unsaturated fatty acids, to maintain membrane stability during stressful conditions in alcoholic fermentations such as low acidity and increasing ethanol concentration. To prevent ethanol shock during fermentation, yeast must alter its cell membrane composition, and the presence of unsaturated fatty acids in the medium proves highly beneficial in this process [21]. Among amino acid metabolism, neither *L. thermotolerans* nor *Hyp. pseudoburtonii* showed a positive correlation with the biosynthesis of aromatic acids, as previously reported by Boban et al. [45] but it is evident that they utilized fatty acids for biomass development, as these two yeasts, along with *P. kluyveri*, achieved the highest average cell unit concentrations during fermentation, specifically 11.68 log CFU/mL for *L. thermotolerans* and 11.16 log CFU/mL for *Hyp. pseudoburtonii*. Truly, the yeast isolates exhibited different fermentation dynamics, which were detailed daily in our previous study [32] through monitoring the viability of yeast cells and tracking the basic physicochemical parameters of the wine. Briefly, *M. pulcherrima* required 40 days to complete fermentation, while *M. chrysoperlae* finished the process the fastest, within 18 days. Interestingly, the yeasts achieved ethanol levels ranging from 11.78% *v*/*v* (*H. pseudoguilliermondii*) to 12.74% *v*/*v* (*M. sinensis/shanxiensis*), demonstrating their resilience under the stressful fermentation conditions and showing fermentation power as they were the only yeasts present at the end of fermentation, due to the use of sterile grape juice. This resilience was likely supported by lipid utilization, as mentioned above. Total acidity varied depending on the yeast isolate compared to the *S. cerevisiae* control, generally resulting in lower acidity. The lowest acidity was observed in the fermentation with *H. uvarum* isolate (5.97 g/L), although the pH value remained statistically similar across samples [32].

The concentration of 1-linoleoyl-rac-GL in monoculture fermentations was increased by *M. chrysoperlae* (0.43 mg/L)*, M. pulcherrima* (0.33 mg/L), and *L. thermotolerans* (0.35 mg/L), but without significant impact compared to the initial concentration (0.29 mg/L). Conversely, fermentations with *P. kluyveri* (0.16 mg/L) and *Hyp. pseudoburtonii* (0.24 mg/L) resulted in significantly lower concentrations than *S. cerevisiae* fermentation trials (0.43 mg/L). Monoacylglycerols in wine, including 1-linoleoyl-rac-GL, are typically the focus of sparkling wine analysis, representing one of the most abundant lipid groups [46]. However, they may enhance the mouthfeel and texture of the wine as active compounds on its surface, aiding in the promotion and stabilization of sparkling wine foam [47].

*H. pseudoguilliermondii* had the most significant effect on the concentration of free fatty esters. The application of this yeast resulted in the highest concentrations of ethyl palmitate (0.73 mg/L), ethyl linoleate (0.33 mg/L), and ethyl oleate (1.29 mg/L), which significantly differed from the concentrations in grape juice and control wines with *S. cerevisiae.* Esters are mostly produced by yeast metabolism of the fatty acid acyl-Coenzyme A (CoA) and acetyl-CoA pathways where CoA serves as a cofactor in metabolic processes- activating intermediates during the biosynthesis of medium-chain fatty acids. The resulting acyl-CoA intermediates are subsequently esterified with ethanol through the action of esterase and transferase enzymes, leading to the formation of fatty ethyl esters. Ethyl esters contribute to the complexity of wine aromas and flavors, though they are typically present at relatively low concentrations [48]. Their role is primarily linked to enhancing the overall mouthfeel and contributing to the aromatic profile, particularly in terms of fruity and waxy characteristics [1]. Additionally, *P. kluyveri* resulted in the lowest concentration of methyl oleate (0.18 mg/L), down from its initial concentration of 0.44 mg/L. When comparing *M. pulcherrima* with the commercial control strain *M. pulcherrima* Flavia, differences were only observed in the metabolizing of ethyl stearate, with the indigenous strain finishing with a lower concentration. Seguinot et al. [49] observed that fermentations involving *M. pulcherrima* produced low levels of acetic acid, likely due to the limited availability of acetyl-CoA. This, in turn, reduces the production of fatty acids and their associated esters. Truly, the indigenous *M. pulcherrima* produced low levels of acetic acid previously reported by Boban et al. [32]. However, we did not notice a link between low levels of acetic acid and a reduction in fatty acids and esters suggesting a biosynthesis of fatty acid more prevalent. As for the control fermentation with *L. thermotolerans* Octave, no significant differences were found compared to the indigenous *L. thermotolerans* strain.

#### 3.2.2. Sequential Fermentations

Indigenous *L. thermotolerans* in sequential fermentations with *S. cerevisiae* produced significantly lower concentrations of arachidic acid (0.38 mg/L), stearic acid (8.71 mg/L), and palmitic acid (17.89 mg/L) compared to control fermentations with *S. cerevisiae*. Varela et al. [50] reported that the metabolism of stearic, oleic, and palmitic acid stimulates the production of the esters, higher alcohols, and volatile fatty acids of wine. Furthermore, *M. sinensis/shanxiensis* and *H. uvarum* in sequential fermentation resulted in statistically lower concentrations of myristic acid, while *M. chrysoperlae×S. cerevisiae* resulted in a lower concentration of stearic acid (8.36 mg/L) compared to *S. cerevisiae.* Zara et al. [51] found a correlation between the lipid composition of yeast cells, the expression of key genes involved in lipid metabolism, and the fermentative performance of wine yeast strains. Specifically, a positive relationship was identified between strong fermentative ability, elevated fatty acid content, and the expression of the ACC1 gene, which encodes a protein essential for the de novo biosynthesis of long-chain fatty acids. Among free fatty acids, the only significant change from the initial concentration was observed for erucic acid (0.03 mg/L), which decreased to 0.01 mg/L in all fermentations, and linoleic acid (0.24 mg/L), which decreased to 0.19 mg/L in *P. kluyveri* × *S. cerevisiae* ferments. Linoleic acid is released into grape juice, where it acts as a substrate for lipoxygenase and hydroxyperoxide lyase activities. These enzymes are responsible for forming C6-aldehydes and alcohols, which contribute to the green flavor [52]. Unsaturated free fatty acids are essential for yeast under anaerobic conditions to maintain membrane integrity and better withstand environmental conditions in alcoholic fermentation [53]. This finding aligns with our previous research, which demonstrated that the *P. kluyveri* isolate, when used in sequential fermentation with *S. cerevisiae*, produced the highest concentrations of phenylacetaldehyde and 2-phenylethanol compared to the other six yeasts studied [32]. Oleic acid is effective in mitigating the toxic effect of ethanol, as its incorporation into lipid membranes leads to a compensatory decrease in membrane fluidity [54]. In this study, all yeast strains reduced the concentration of oleic acid during alcoholic fermentation. Liu et al. [55] demonstrated that even minor fluctuations in unsaturated fatty acid concentrations can greatly affect the aromatic profile of wines. Baron et al. [56] extensively studied the inhibitory effect of medium-chain fatty acids (MCFAs) on yeasts, finding that a dose of 10 mg/L had toxic effects on *S. cerevisiae*. Licek et al. [57] further explored how an MCFA mixture influences the levels of carbonyl compounds in wine during the final stages of alcoholic fermentation. The concentrations of total free fatty acid significantly differed between indigenous yeasts, *M. chrysoperlae, L. thermotolerans, H. uvarum,* and *H. guilliermondii,* in sequential fermentations compared to control *S. cerevisiae* fermentations with lower concentrations. Furthermore, sequential fermentations with indigenous *L. thermotolerans* differed from those with the commercial *L. thermotolerans* Octave. Ochando et al. [19] observed that fatty acids and sterols positively contribute to glycerol production. In our previous research [32], it was reported that *H. guilliermondii* and *L. thermotolerans* in sequential fermentation demonstrated the fastest fermentation kinetics, completing the process within 16 days, while *M. chrysoperlae × S. cerevisiae* required 24 days. This fatty acid utilization possibly led to the excellent competitiveness of *L. thermotolerans and H. guilliermondii* ending fermentation with higher cell concentrations compared to *S. cerevisiae* [32]. Additionally, weaker utilization of lipid components led to the dominance of the killer effect of *S. cerevisiae*, resulting in *M. pulcherrima* being absent from the wine on the final day of fermentation (12.35% *v*/*v* of ethanol) and *H. uvarum* being absent during the last four days (10.54% *v*/*v* of ethanol), leaving *S. cerevisiae* as the sole yeast present while other yeast isolates demonstrated excellent resilience [32]. Briefly, regarding basic physicochemical parameters, the lowest ethanol concentration was produced by *M. sinensis/shanxiensis* (11.91% *v*/*v*), while *H. guilliermondii* produced the highest (12.51% *v*/*v*). In terms of total acidity, no significant impact was observed. However, the pH value of the *H. uvarum × S. cerevisiae* fermentation (3.33) was significantly higher than that of the other fermentations and the *S. cerevisiae* control (3.25) [32].

The concentration of 1-linoleoyl-rac-GL increased during alcoholic fermentation with *S. cerevisiae* which exhibited higher concentrations compared to other fermentations. In contrast, sequential fermentations with *M. chrysoperlae, M. sinensis/shanxiensis, H. uvarum, H. guilliermondii*, and *P. kluyveri* showed a decrease in 1-linoleoyl-rac-GL concentrations, which were statistically different compared to the *S. cerevisiae* monoculture control fermentation (0.43 mg/L). Additionally, indigenous *M. pulcherrima* and *L. thermotolerans* produced statistically higher concentrations of 1-linoleoyl-rac-GL compared to their respective commercial strains, *L. thermotolerans* Octave and *M. pulcherrima* Flavia. While the initial release of lipids into the wine matrix is rapid, their concentration tends to decrease over time due to the esterification of monoacylglycerol, diacylglycerol, and triacylglycerol [46]. However, *L. thermotolerans* in sequential fermentations was the only indigenous yeast to increase 1-linoleoyl-rac-GL, indicating that no esterification of monoacylglycerol occurred in these fermentations.

Even in this study, although differences were observed among fatty acids and their corresponding ethyl esters, there was only one significant difference in the concentration of methyl oleate. During fermentation, yeast may produce esters to compensate for disruptions in the CoA-SH/acetyl-CoA ratio that arise from the lipid synthesis halt [58]. Both *M. sinensis/shanxiensis × S. cerevisiae* and *H. guilliermondii × S. cerevisiae* exhibited statistically lower concentrations of methyl oleate compared to the *S. cerevisiae* control. Additionally, there was a decrease in its concentration across all fermentations compared to the initial concentration in Maraština grape juice (0.44 mg/L). Tai et al. [59] compared various genome-wide transcriptional studies of *S. cerevisiae* grown at low temperatures and concluded that only genes involved in lipid metabolism were regulated in response to low-temperature conditions.

### 3.3. Hierarchical Clustering and Pathway Analyses

**Figure 1:**
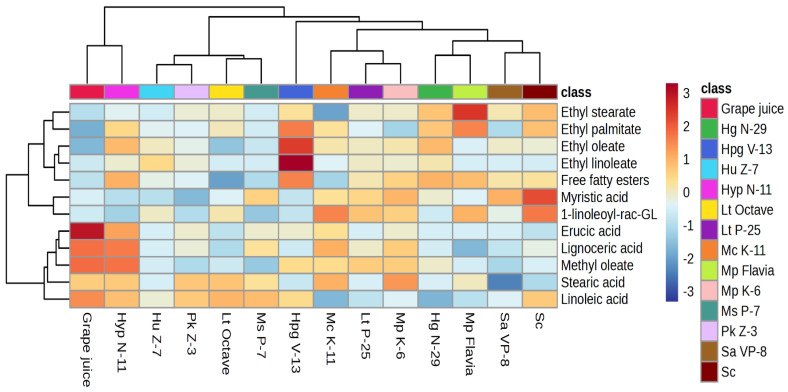
Hierarchical clustering analysis was performed on the significant lipid compounds in grape juice and across 10 monoculture fermentations with indigenous non-*Saccharomyces* yeasts, as well as three control fermentations (*S. cerevisiae*, *L. thermotolerans* Octave, and *M. pulcherrima* Flavia). The rows represent the lipid compounds, while the columns display the different fermentation trials. The color scale indicates the abundance of compounds in the samples, ranging from dark blue (minimum) to dark red (maximum). 1-linoleoyl-rac-GL—1-linoleoyl-rac-glycerol; *Sc—S. cerevisiae.*

**Figure 2:**
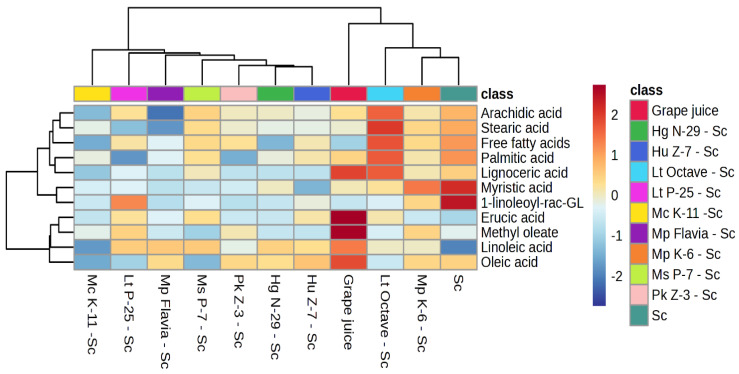
Hierarchical clustering analysis was performed on the significant lipid compounds in grape juice and across seven sequential fermentations with indigenous non-*Saccharomyces* yeasts and *S. cerevisiae*, as well as three control fermentations (*S. cerevisiae*, *L. thermotolerans* Octave-*S. cerevisiae*, and *M. pulcherrima* Flavia-*S. cerevisiae*). The rows represent the lipid compounds, while the columns display the different fermentation trials. The color scale indicates the abundance of compounds in the samples, ranging from dark blue (minimum) to dark red (maximum). 1-linoleoyl-rac-GL—1-linoleoyl-rac-glycerol; *Sc—S. cerevisiae.*

Hierarchical clustering analyses were performed on two data sets obtained from monoculture fermentations (Figure 1) and sequential fermentations (Figure 2). The heatmap was generated using components that showed statistically significant differences in lipid concentrations. Figure 1 visualized differences between yeasts in monoculture where yeast can be classified into five groups, as follows: (i) *Hyp. pseudoburtonii* and grape juice, (ii) *H. uvarum*, *P. kluyveri, L. thermotolerans* Octave, and *M. sinensis/shanxiensis*, (iii) *H. pseudoguilliermondii*, (iv) *M. chrysoperlae, L. thermotolerans,* and *M. pulcherrima,* and (v) *H. guilliermondii*, *M. pulcherrima* Flavia, *Starmerella apicola,* and *S. cerevisiae. M. pulcherrima* and *L. thermotolerans* grouped different forms of their commercial controls Flavia and Octave. Furthermore, *M. chrysoperlae* and *M. pulcherrima* originated from the same vineyard [31] and grouped, also with *H. uvarum* and *P. kluyveri* possibly due to the terroir impact on yeast characteristics given that they had similar lipase activity previously reported by [33]. Lipases are versatile enzymes that facilitate a broad spectrum of reactions, such as hydrolysis, inter-esterification, alcoholysis, acidolysis, esterification, and aminolysis. Their primary function is to catalyze the hydrolysis of fatty acid ester bonds in triacylglycerols, resulting in the release of free fatty acids [60]. Conversely, the same origin is for *Hyp. pseudoburtonii* and *H. guilliermondii,* and for *M. sinensis/shanxiensis* and *L. thermotolerans* but we did not find a relation with vineyards where the yeast strain has the upper hand.

Figure 2 pointed out clustering in sequential fermentation. *M. chrysoperlae*, *L. thermotolerans*, M. sinensis/shanxiensis, *P. kluyveri*, *H. guilliermondii*, *H. uvarum*, and *M. pulcherrima* Flavia build the first group while two commercial strains, *L. thermotolerans* Octave, S. cerevisiae EC 1118, and *M. pulcherrima*, and initial concentrations in grape juice formed a second group. All yeasts with grape juice were classified into two groups highlighting the impact of S. cerevisiae EC 1118 which uniform the lipid content in his study, and in general wine characteristics [61].

**Figure 3:**
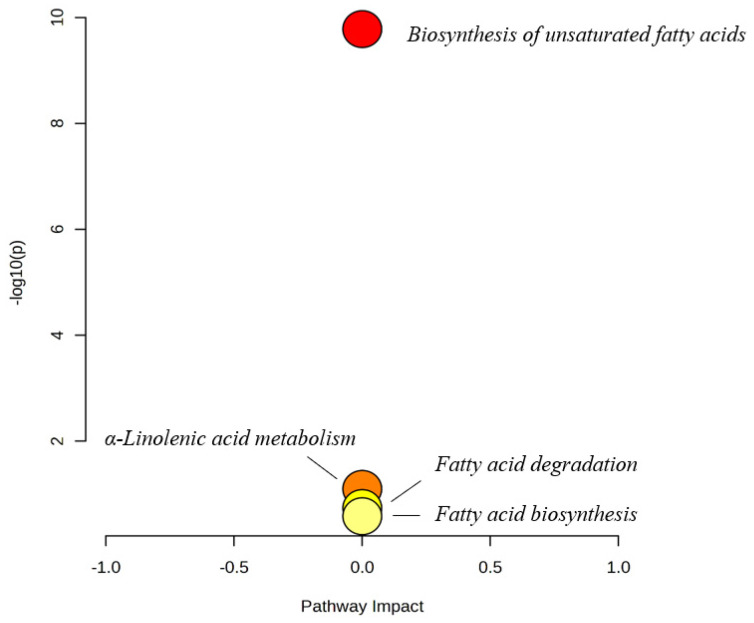
Pathway analysis was performed using all quantified metabolites with the Kyoto Encyclopedia of Genes and Genomes (KEGG) IDs, utilizing *Saccharomyces cerevisiae* as the reference pathway library from KEGG. The color gradient represents *p*-values, with yellow indicating lower values and red indicating higher values.

One metabolic pathway particularly stood out compared with other pathways with its impact (shown in the *x*-axis in Figure 3) and significance (shown in the *y*-axis in Figure 3). The *x*-axis represents the “Pathway Impact,” which quantifies the importance or relevance of a specific metabolic pathway based on its topology analysis. The further right a point is, the more impactful the pathway is. The *y*-axis represents ${-log}_{10}\left(p\right),$ which corresponds to the statistical significance of the pathway (usually derived from enrichment analysis). Biosynthesis of unsaturated fatty acids pathways are marked in red, while other pathways are clustered near zero on the impact scale and shown in yellow to orange colors, which could be traced back to the presence of relatively higher concentrations of unsaturated fatty acids in the wines.

### 3.4. Comparisons of Fermentation Practices with Non-Saccharomyces Isolates

In the previous section, the impact of yeasts in monoculture and sequential fermentation on the lipid composition in wine and lipid changes was discussed. However, when comparing the initial concentration of lipid compounds in Maraština grape juice with their concentrations in the resulting wines, certain trends of concentration increase or decrease can be observed for specific compounds, regardless of the inoculation method used. This section will focus on the opposing effects of fermentation practices: certain fermentation practices increase the concentration of a particular compound while others decrease it compared to the initial composition, even when using the same non-*Saccharomyces* isolate. This effect was particularly noticeable with free fatty acids such as arachidic acid, myristic acid, stearic acid, palmitic acid, oleic acid, and palmitoleic acid, where the reaction of degradation or synthesis varied depending on whether the yeast was used in monoculture or sequential fermentation. A similar pattern was observed with oleanolic acid, 1-linoleoyl-rac-GL, ethyl palmitate, and ethyl oleate, while the other compounds followed consistent trends—either increasing or decreasing—in both fermentation methods, as previously discussed.

More specifically, *M. chrysoperlae* showed a higher production of all five total lipid groups in monoculture (∑Free fatty acids, ∑Triterpenoids, ∑Glycerolipids, ∑Free fatty esters). Stearic acid, palmitic acid, palmitoleic acid, oleanolic acid, and 1-linoleoyl-rac-GL were increased in monoculture fermentations, while sequential fermentations led to a reduction in their concentrations compared to the initial levels. The utilization of lipid compounds in sequential fermentations is one of the reasons why *M. chrysoperlae* produced more esters and higher alcohols during sequential fermentation compared to monoculture, as reported in a previous study [32]. *M. sinensis/shanxiensis* exhibited a different pattern, with an increase in stearic acid and oleanolic acid during sequential fermentation, while myristic and palmitoleic acid concentrations decreased compared to the start of fermentation. Additionally, *M. sinensis/shanxiensis* was the only yeast that produced more fatty esters in sequential fermentation than in monoculture. Incorporating stearic acid into cell membranes is one way yeast responds to ethanol resistance, which in turn decreases the stearic acid content in wine. This suggests that *M. sinensis/shanxiensis* has a higher demand for lipids in monoculture than in sequential fermentation, where *S. cerevisiae* plays the primary role in completing alcoholic fermentation [21]. The third yeast from the *Metschnikowia* genus, *M. pulcherrima*, showed results more aligned with *M. chrysoperlae*, where monoculture fermentation led to increased concentrations of palmitic acid, 1-linoleoyl-rac-GL, total free fatty acids, and esters. However, the production of stearic, palmitoleic, and oleanolic acids favored sequential fermentation with *L. thermotolerans* and *S. cerevisiae* by increasing concentrations of these compounds, rather than monoculture with *L. thermotolerans*, suggesting that indigenous *L. thermotolerans* had higher fatty acid requirements due to the stressful conditions of alcoholic fermentation [21]. Both yeasts from the *Hanseniaspora* genus showed similar patterns in lipid synthesis or degradation and exhibited very similar behaviors in both monoculture and sequential fermentations. For example, *H. uvarum* only differed in oleanolic acid, which increased in monoculture and decreased in sequential fermentations, while the reverse was true for stearic acid. Interestingly, the cell of *H. uvarum* was not detected at the end of sequential fermentations [27] which aligns with the fact that stearic acid was produced during this fermentation and not utilized. Most of the identified compounds were metabolized to lower concentrations than those in the grape juice, except for the esters, except for methyl oleate. Notably, methyl oleate decreased in all alcoholic fermentations across all trials. *H. guilliermondii* also reduced all lipid components in both fermentation practices, except for esters and palmitoleic acid, which increased during sequential fermentation compared to the initial concentration in grape juice. Lastly, *P. kluyveri* followed a similar pattern, except for arachidic and oleic acid, which favored monoculture fermentation, while ethyl oleate favored sequential fermentation, resulting in increased concentrations due to the initial one.

Considering that the wine production process is not sterile, the diversity of microorganisms can influence the lipid profile through complex interactions between non-*Saccharomyces* and *Saccharomyces* yeasts, as well as other microorganisms. Non-*Saccharomyces* yeasts, such as *Hanseniaspora*, *P. kluyveri*, and *M. chrysoperlae*, have shown a significant impact on specific lipid compounds, including reductions in lignoceric acid, free fatty esters, and 1-linoleoyl-rac-GL. Sequential fermentation further alters lipid profiles, with some yeasts enhancing ester production or modifying free fatty acid concentrations. Such responses are amplified under real non-sterile conditions, resulting in diverse lipid dynamics that contribute to unique aroma profiles and wine complexity.

## 4. Conclusions

Lipids are a crucial component of grapes, impacting fermentation processes and influencing wine characteristics as essential nutrients and precursors of aroma compounds. In monoculture fermentations, yeast strains from the *Hanseniaspora* genera significantly reduced lignoceric acid, while *H. pseudoguilliermondii* affected free fatty esters. Additionally, *P. kluyveri* and *Hyp. pseudoburtonii* reduced 1-linoleoyl-rac-GL compared to the *S. cerevisiae* control and affected the concentration of free fatty acids as well as *M. chrysoperlae* and *M. pulcherrima*. Furthermore, sequential fermentation was observed for seven non-*Saccharomyces* isolates with enhanced enzymatic activity: *M. sinensis/shanxiensis, M. chrysoperlae, M. pulcherrima*, *L. thermotolerans*, *H. uvarum*, *H. guilliermondii*, and *P. kluyveri*. Sequential fermentations with *M. chrysoperlae*, *M. sinensis/shanxiensis*, *H. uvarum*, *H. guilliermondii*, and *P. kluyveri* showed a decrease in 1-linoleoyl-rac-GL concentrations, which were statistically different compared to the *S. cerevisiae* monoculture control. Sequential fermentations did not significantly affect free fatty esters, except in *M. sinensis/shanxiensis × S. cerevisiae* and *H. guilliermondii × S. cerevisiae* fermentations for methyl oleate. Certain components exhibit an opposite trend (increasing or decreasing) in sequential fermentation and monoculture compared to the initial must. *M. chrysoperlae* and *M. pulcherrima* favor monoculture for the production of total free fatty acids and esters, while *M. sinensis/shanxiensis* produces more esters in sequential fermentation with *S. cerevisiae*. The *Hanseniaspora* genus was the yeast least affected by the inoculation treatment, with almost all compounds following the same trend. The exception for *H. uvarum* was in oleanolic and stearic acid, and for *H. guilliermondii* was palmitoleic acid. Hierarchical clustering analysis grouped yeast isolates in both monoculture and sequential fermentations based on their influence on the synthesis or degradation of lipid compounds. The most notable impact in this study was observed in the biosynthesis of the unsaturated fatty acid pathway.

## Figures and Tables

**Table 1:** Concentration (mg/L) of lipid compounds in grape juice and Maraština wines produced for ten indigenous non-Saccharomyces yeast isolates and three commercial yeasts (controls): *S. cerevisiae* EC 1118, *L. thermotolerans* Octave, and *M. pulcherrima* Flavia.

Compounds	Grape Juice	*Hyp. pseudoburtonii* N-11	*M. chrysoperlae* K-11	*M. sinesis/* *shanxiensis* P-7	*M.* *pulcherrima* K-6	*L. thermotolerans* P-25	*H.* *uvarum* Z-7	*H. guilliermondii* N-29	*H. pseudoguilliermondii* V-13	*P.* *kluyveri* Z-3	*S.* *apicola* VP-8	*S.* *cerevisiae* EC 1118	*L. thermotolerans* Octave	*M. pulcherrima* Flavia
Lignoceric acid	0.57 ± 0.03 ^d^	0.55 ± 0.08 ^cd^	0.47 ± 0.09 ^bcd^	0.37 ± 0.07 ^abcd^	0.41 ± 0.11 ^abcd^	0.32 ± 0.12 ^abc^	0.25 ± 0.12 ^ab^	0.25 ± 0.03 ^ab^	0.24 ± 0.02 ^ab^	0.30 ± 0.08 ^ab^	0.30 ± 0.05 ^abcd^	0.35 ± 0.13 ^abcd^	0.24 ± 0.04 ^ab^	0.22 ± 0.03 ^a^
Behenic acid	0.13 ± 0.02	0.10 ± 0.03	0.10 ± 0.05	0.07 ± 0.01	0.09 ± 0.04	0.06 ± 0.02	0.05 ± 0.02	0.05 ± 0.01	0.05 ± 0.01	0.11 ± 0.03	0.06 ± 0.01	0.09 ± 0.05	0.05 ± 0.01	0.05 ± 0.01
Arachidic acid	0.38 ± 0.01	0.38 ± 0.05	0.38 ± 0.07	0.34 ± 0.01	0.37 ± 0.08	0.31 ± 0.04	0.34 ± 0.00	0.33 ± 0.02	0.32 ± 0.02	0.41 ± 0.03	0.35 ± 0.01	0.43 ± 0.08	0.33 ± 0.02	0.33 ± 0.02
Myristic acid	0.56 ± 0.29 ^ab^	0.69 ± 0.32 ^ab^	0.55 ± 0.18 ^ab^	0.59 ± 0.12 ^ab^	0.65 ± 0.17 ^ab^	0.58 ± 0.21 ^ab^	0.52 ± 0.03 ^ab^	0.49 ± 0.08 ^ab^	0.39 ± 0.04 ^ab^	0.27 ± 0.07 ^a^	0.66 ± 0.01 ^ab^	0.83 ± 0.18 ^b^	0.45 ± 0.10 ^ab^	0.45 ± 0.11 ^ab^
Stearic acid	8.64 ± 0.43 ^ab^	8.69 ± 0.77 ^ab^	8.94 ± 0.91 ^ab^	8.42 ± 1.91 ^ab^	9.15 ± 1.92 ^ab^	7.88 ± 0.51 ^a^	7.86 ± 1.24 ^ab^	7.87 ± 0.79 ^a^	7.74 ± 0.42 ^ab^	8.71 ± 0.71 ^ab^	9.09 ± 0.84 ^ab^	10.87 ± 0.47 ^b^	8.74 ± 0.22 ^ab^	8.23 ± 0.25 ^ab^
Palmitic acid	18.9 ± 0.70	19.28 ± 1.33	19.71 ± 2.18	18.05 ± 1.50	19.25 ± 2.95	16.82 ± 1.88	18.62 ± 2.09	17.56 ± 0.68	19.03 ± 0.28	16.52 ± 2.62	18.85 ± 2.95	21.09 ± 2.91	17.08 ± 0.67	16.77 ± 0.59
Erucic acid	0.03 ± 0.00 ^c^	0.02 ± 0.01 ^b^	0.01 ± 0.00 ^ab^	0.01 ± 0.00 ^ab^	0.01 ± 0.01 ^ab^	0.01 ± 0.00 ^ab^	0.01 ± 0.00 ^ab^	0.01 ± 0.00 ^ab^	0.01 ± 0.01 ^ab^	0.01 ± 0.01 ^ab^	0.01 ± 0.00 ^ab^	0.01 ± 0.00 ^a^	0.01 ± 0.00 ^a^	0.01 ± 0.00 ^a^
Linolenic acid	0.06 ± 0.01	0.06 ± 0.01	0.05 ± 0.01	0.04 ± 0.00	0.04 ± 0.00	0.05 ± 0.01	0.06 ± 0.01	0.05 ± 0.01	0.06 ± 0.02	0.05 ± 0.01	0.04 ± 0.00	0.04 ± 0.00	0.04 ± 0.00	0.04 ± 0.00
Linoleic acid	0.24 ± 0.01 ^b^	0.21 ± 0.00 ^ab^	0.21 ± 0.02 ^ab^	0.22 ± 0.01 ^ab^	0.21 ± 0.01 ^ab^	0.19 ± 0.01 ^a^	0.22 ± 0.04 ^ab^	0.21 ± 0.01 ^ab^	0.19 ± 0.01 ^ab^	0.20 ± 0.01 ^ab^	0.21 ± 0.02 ^ab^	0.20 ± 0.01 ^ab^	0.21 ± 0.01 ^ab^	0.18 ± 0.02 ^a^
Oleic acid	1.18 ± 0.08	1.07 ± 0.18	1.07 ± 0.14	0.95 ± 0.15	1.05 ± 0.09	1.00 ± 0.12	0.91 ± 0.03	0.95 ± 0.05	0.99 ± 0.03	1.21 ± 0.68	0.93 ± 0.04	0.94 ± 0.09	0.91 ± 0.11	0.91 ± 0.03
Palmitoleic acid	0.16 ± 0.04	0.09 ± 0.01	0.22 ± 0.08	0.25 ± 0.28	0.11 ± 0.01	0.12 ± 0.10	0.11 ± 0.01	0.30 ± 0.13	0.32 ± 0.08	0.06 ± 0.04	0.27 ± 0.04	0.14 ± 0.02	0.05 ± 0.03	0.07 ± 0.04
∑Free fatty acids	30.84 ± 0.91	31.12 ± 2.62	31.72 ± 3.43	29.31 ± 3.72	31.35 ± 5.12	27.33 ± 2.81	28.94 ± 3.41	28.06 ± 1.62	29.34 ± 0.26	27.85 ± 4.13	30.77 ± 2.02	35.00 ± 3.18	28.11 ± 0.62	27.27 ± 0.87
Oleanolic acid	0.05 ± 0.01	0.05 ± 0.02	0.08 ± 0.05	0.03 ± 0.02	0.05 ± 0.04	0.04 ± 0.01	0.06 ± 0.03	0.04 ± 0.01	0.04 ± 0.01	0.05 ± 0.01	0.06 ± 0.02	0.09 ± 0.04	0.37 ± 0.55	0.04 ± 0.01
∑Triterpenoids	0.05 ± 0.01	0.05 ± 0.02	0.08 ± 0.05	0.03 ± 0.02	0.05 ± 0.04	0.04 ± 0.01	0.06 ± 0.03	0.04 ± 0.01	0.04 ± 0.01	0.05 ± 0.01	0.06 ± 0.02	0.09 ± 0.04	0.37 ± 0.55	0.04 ± 0.01
1-linoleoyl-rac-GL	0.29 ± 0.02 ^abc^	0.24 ± 0.06 ^ab^	0.43 ± 0.10 ^bc^	0.27 ± 0.06 ^abc^	0.33 ± 0.03 ^abc^	0.35 ± 0.04 ^abc^	0.26 ± 0.03 ^abc^	0.29 ± 0.03 ^abc^	0.27 ± 0.04 ^abc^	0.16 ± 0.01 ^a^	0.24 ± 0.02 ^abc^	0.43 ± 0.12 ^c^	0.37 ± 0.07 ^bc^	0.37 ± 0.10 ^bc^
∑Glycerolipids	0.29 ± 0.02 ^abc^	0.24 ± 0.06 ^ab^	0.43 ± 0.10 ^bc^	0.27 ± 0.06 ^abc^	0.33 ± 0.03 ^abc^	0.35 ± 0.04 ^abc^	0.26 ± 0.03 ^abc^	0.29 ± 0.03 ^abc^	0.27 ± 0.04 ^abc^	0.16 ± 0.01 ^a^	0.24 ± 0.02 ^abc^	0.43 ± 0.12 ^c^	0.37 ± 0.07 ^bc^	0.37 ± 0.10 ^bc^
1,2-dioleoyl_GLP_Na	0.01 ± 0.01	0.01 ± 0.02	0.00 ± 00	0.01 ± 0.01	0.00 ± 00	0.00 ± 00	0.00 ± 00	0.00 ± 00	0.00 ± 00	0.01 ± 0.01	0.00 ± 00	0.00 ± 00	0.00 ± 00	0.00 ± 00
∑Glyceropholipids	0.01 ± 0.01	0.01 ± 0.02	0.00 ± 00	0.01 ± 0.01	0.00 ± 00	0.00 ± 00	0.00 ± 00	0.00 ± 00	0.00 ± 00	0.01 ± 0.01	0.00 ± 00	0.00 ± 00	0.00 ± 00	0.00 ± 00
Methyl oleate	0.44 ± 0.04 ^b^	0.44 ± 0.06 ^b^	0.43 ± 0.03 ^b^	0.28 ± 0.17 ^ab^	0.33 ± 0.02 ^ab^	0.33 ± 0.05 ^ab^	0.23 ± 0.13 ^ab^	0.27 ± 0.01 ^ab^	0.31 ± 0.01 ^ab^	0.18 ± 0.04 ^a^	0.27 ± 0.05 ^ab^	0.24 ± 0.04 ^a^	0.22 ± 0.03 ^a^	0.23 ± 0.02 ^a^
Ethyl palmitate	0.44 ± 0.04 ^a^	0.58 ± 0.09 ^abc^	0.56 ± 0.08 ^ac^	0.45 ± 0.11 ^abc^	0.55 ± 0.08 ^abc^	0.47 ± 0.07 ^abc^	0.48 ± 0.09 ^abc^	0.61 ± 0.07 ^abc^	0.73 ± 0.16 ^bc^	0.47 ± 0.11 ^abc^	0.57 ± 0.04 ^abc^	0.63 ± 0.09 ^abc^	0.53 ± 0.01 ^abc^	0.72 ± 0.05 ^b^
Methyl stearate	n.d.	0.06 ± 0.04 ^ab^	0.05 ± 0.03 ^a^	0.02 ± 0.03 ^a^	0.18 ± 0.09 ^b^	0.13 ± 0.06 ^ab^	0.12 ± 0.00 ^ab^	0.15 ± 0.03 ^ab^	0.16 ± 0.06 ^ab^	0.17 ± 0.02 ^b^	0.15 ± 0.01 ^ab^	0.10 ± 0.03 ^ab^	0.10 ± 0.01 ^ab^	0.11 ± 0.01 ^ab^
Ethyl linoleate	0.04 ± 0.00 ^a^	0.14 ± 0.08 ^ab^	0.06 ± 0.01 ^a^	0.05 ± 0.01 ^a^	0.08 ± 0.01 ^a^	0.09 ± 0.04 ^a^	0.12 ± 0.10 ^ab^	0.09 ± 0.05 ^a^	0.33 ± 0.26 ^b^	0.08 ± 0.02 ^a^	0.05 ± 0.00 ^a^	0.05 ± 0.00 ^a^	0.05 ± 0.00 ^a^	0.05 ± 0.00 ^a^
Ethyl oleate	0.32 ± 0.02 ^a^	0.60 ± 0.27 ^a^	0.49 ± 0.11 ^a^	0.36 ± 0.13 ^a^	0.49 ± 0.03 ^a^	0.47 ± 0.13 ^a^	0.47 ± 0.07 ^a^	0.59 ± 0.03 ^a^	1.29 ± 0.44 ^b^	0.43 ± 0.07 ^a^	0.46 ± 0.02 ^a^	0.45 ± 0.03 ^a^	0.38 ± 0.01 ^a^	0.41 ± 0.03 ^a^
Ethyl stearate	0.24 ± 0.03 ^a^	0.30 ± 0.03 ^a^	0.33 ± 0.05 ^a^	0.27 ± 0.08 ^a^	0.32 ± 0.06 ^a^	0.32 ± 0.03 ^a^	0.27 ± 0.09 ^a^	0.39 ± 0.07 ^a^	0.35 ± 0.11 ^ab^	0.31 ± 0.07 ^a^	0.33 ± 0.04 ^a^	0.40 ± 0.03 ^ab^	0.32 ± 0.02 ^a^	0.54 ± 0.05 ^b^
∑Free fatty esters	1.49 ± 0.09 ^a^	2.11 ± 0.51 ^a^	1.92 ± 0.26 ^a^	1.42 ± 0.52 ^a^	1.95 ± 0.13 ^a^	1.81 ± 0.18 ^a^	1.69 ± 0.14 ^a^	2.11 ± 0.18 ^a^	3.17 ± 0.38 ^b^	1.64 ± 0.28 ^a^	1.84 ± 0.15 ^a^	1.86 ± 0.20 ^a^	1.59 ± 0.06 ^a^	2.06 ± 0.07 ^a^

The value of volatile compounds is expressed as mean ± standard deviation (n = 3). Different letters in the column indicate a significant difference (*p* < 0.05). Abbreviations: 1,2-dioleoyl_GLP_Na—1,2-dioleoyl-sn-glycero-3-phospho-rac-(1-glycerol) sodium salt; 1linoleoyl-rac-GL—1-linoleoyl-rac glycerol.

**Table 2:** Concentration (mg/L) of lipid compounds in grape juice and Maraština wines produced for seven indigenous non-*Saccharomyces* yeast isolates and two commercial yeasts: *L. thermotolerans* Octave and *M. pulcherrima* Flavia in sequential fermentation with *S. cerevisiae* and in pure fermentation with *S. cerevisiae*.

Compound	Grape Juice	*M. chrysoperlae* K-11—*Sc*	*M. sinensis/shanxiensis* P-7—*Sc*	*M. pulcherrima* K-6—*Sc*	*L. thermotolerans* P-25—*Sc*	*H. uvarum* Z-7—*Sc*	*H. guilliermondii* N-29—*Sc*	*P. kluyveri* Z-3—*Sc*	*Sc*	*L.* *thermotolerans* Octave—*Sc*	*M. pulcherrima* Flavia—*Sc*
Lignoceric acid	0.57 ± 0.03 ^c^	0.15 ± 0.04 ^a^	0.28 ± 0.07 ^ab^	0.28 ± 0.04 ^ab^	0.32 ± 0.03 ^abc^	0.26 ± 0.06 ^ab^	0.23 ± 0.08 ^a^	0.21 ± 0.08 ^a^	0.35 ± 0.13 ^abc^	0.54 ± 0.22 ^bc^	0.21 ± 0.08 ^a^
Behenic acid	0.13 ± 0.02	0.04 ± 0.02	0.09 ± 0.02	0.07 ± 0.02	0.09 ± 0.02	0.07 ± 0.03	0.07 ± 0.03	0.06 ± 0.05	0.09 ± 0.05	0.12 ± 0.02	0.06 ± 0.02
Arachidic acid	0.38 ± 0.01 ^ab^	0.32 ± 0.02 ^a^	0.39 ± 0.04 ^ab^	0.36 ± 0.04 ^a^	0.38 ± 0.04 ^a^	0.33 ± 0.06 ^a^	0.35 ± 0.04 ^a^	0.35 ± 0.06 ^a^	0.43 ± 0.08 ^ab^	0.51 ± 0.05 ^b^	0.34 ± 0.01 ^a^
Myristic acid	0.56 ± 0.29 ^ab^	0.38 ± 0.16 ^ab^	0.35 ± 0.11 ^a^	0.70 ± 0.19 ^ab^	0.40 ± 0.10 ^ab^	0.28 ± 0.04 ^a^	0.45 ± 0.14 ^ab^	0.37 ± 0.05 ^ab^	0.83 ± 0.18 ^b^	0.50 ± 0.07 ^ab^	0.33 ± 0.05 ^a^
Stearic acid	8.64 ± 0.43 ^ab^	8.36 ± 1.14 ^a^	9.54 ± 0.58 ^ab^	9.71 ± 0.64 ^ab^	8.71 ± 0.93 ^ab^	8.40 ± 0.35 ^ab^	8.41 ± 1.31 ^ab^	8.71 ± 0.53 ^ab^	10.87 ± 0.47 ^bc^	13.23 ± 1.48 ^c^	7.30 ± 0.47 ^a^
Palmitic acid	18.9 ± 0.70 ^abc^	17.02 ± 1.75 ^ab^	18.40 ± 1.33 ^ab^	17.97 ± 0.41 ^ab^	17.89 ± 0.40 ^ab^	17.97 ± 1.73 ^ab^	17.11 ± 1.30 ^ab^	18.76 ± 0.82 ^abc^	21.09 ± 2.91 ^bc^	22.77 ± 1.37 ^c^	16.61 ± 1.10 ^a^
Erucic acid	0.03 ± 0.00 ^b^	0.01 ± 0.00 ^a^	0.01 ± 0.00 ^a^	0.01 ± 0.00 ^a^	0.01 ± 0.00 ^a^	0.01 ± 0.00 ^a^	0.01 ± 0.00 ^a^	0.01 ± 0.00 ^a^	0.01 ± 0.00 ^a^	0.01 ± 0.01 ^a^	0.01 ± 0.00 ^a^
Linolenic acid	0.06 ± 0.01	0.04 ± 0.02	0.05 ± 0.00	0.04 ± 0.00	0.04 ± 0.00	0.04 ± 0.01	0.04 ± 0.00	0.05 ± 0.01	0.04 ± 0.00	0.06 ± 0.01	0.04 ± 0.01
Linoleic acid	0.24 ± 0.01 ^b^	0.20 ± 0.01 ^ab^	0.21 ± 0.03 ^ab^	0.2 ± 0.01 ^ab^	0.21 ± 0.02 ^ab^	0.21 ± 0.01 ^ab^	0.21 ± 0.01 ^ab^	0.19 ± 0.01 ^a^	0.20 ± 0.01 ^ab^	0.20 ± 0.01 ^ab^	0.21 ± 0.02 ^ab^
Oleic acid	1.18 ± 0.08 ^c^	0.89 ± 0.06 ^a^	0.92 ± 0.04 ^ab^	0.94 ± 0.05 ^ab^	1.01 ± 0.03 ^abc^	0.97 ± 0.07 ^abc^	0.92 ± 0.07 ^ab^	0.93 ± 0.10 ^ab^	0.94 ± 0.09 ^ab^	1.14 ± 0.13 ^bc^	0.92 ± 0.07 ^ab^
Palmitoleic acid	0.16 ± 0.04	0.00 ± 0.00	0.00 ± 0.00	0.00 ± 0.00	0.00 ± 0.00	0.00 ± 0.00	0.00 ± 0.00	0.00 ± 0.00	0.00 ± 0.00	0.00 ± 0.00	0.00 ± 0.00
∑Free fatty acids	30.84 ± 0.91 ^ab^	27.45 ± 3.05 ^a^	30.36 ± 1.95 ^ab^	30.42 ± 0.61 ^ab^	29.23 ± 1.38 ^a^	28.59 ± 2.21 ^a^	27.9 ± 0.98 ^a^	29.78 ± 0.53 ^ab^	35.00 ± 3.18 ^bc^	39.20 ± 2.88 ^c^	26.18 ± 1.14 ^a^
Oleanolic acid	0.05 ± 0.01	0.05 ± 0.05	0.23 ± 0.32	0.05 ± 0.01	0.15 ± 0.19	0.05 ± 0.04	0.04 ± 0.01	0.03 ± 0.02	0.09 ± 0.04	0.09 ± 0.02	0.04 ± 0.03
∑Triterpenoid	0.05 ± 0.01	0.05 ± 0.05	0.23 ± 0.32	0.05 ± 0.01	0.15 ± 0.19	0.05 ± 0.04	0.04 ± 0.01	0.03 ± 0.02	0.09 ± 0.04	0.09 ± 0.02	0.04 ± 0.03
1-linoleoyl-rac-GL	0.29 ± 0.02 ^ab^	0.20 ± 0.03 ^a^	0.21 ± 0.03 ^a^	0.27 ± 0.09 ^ab^	0.34 ± 0.11 ^ab^	0.23 ± 0.06 ^a^	0.19 ± 0.01 ^a^	0.18 ± 0.02 ^a^	0.43 ± 0.12 ^b^	0.21 ± 0.02 ^a^	0.18 ± 0.02 ^a^
∑Glycerolipids	0.29 ± 0.02 ^ab^	0.20 ± 0.03 ^a^	0.21 ± 0.03 ^a^	0.27 ± 0.09 ^ab^	0.34 ± 0.11 ^ab^	0.23 ± 0.06 ^a^	0.19 ± 0.01 ^a^	0.18 ± 0.02 ^a^	0.43 ± 0.12 ^b^	0.21 ± 0.02 ^a^	0.18 ± 0.02 ^a^
1,2-dioleoyl_GLP_ Na	0.01 ± 0.01	0.00 ± 0.00	0.00 ± 0.00	0.00 ± 0.00	0.00 ± 0.00	0.00 ± 0.00	0.00 ± 0.00	0.00 ± 0.00	0.00 ± 0.00	0.00 ± 0.00	0.00 ± 0.00
∑Glyceropholipids	0.01 ± 0.01	0.00 ± 0.00	0.00 ± 0.00	0.00 ± 0.00	0.00 ± 0.00	0.00 ± 0.00	0.00 ± 0.00	0.00 ± 0.00	0.00 ± 0.00	0.00 ± 0.00	0.00 ± 0.00
Methyl oleate	0.44 ± 0.04 ^c^	0.18 ± 0.01 ^ab^	0.16 ± 0.01 ^a^	0.24 ± 0.03 ^b^	0.24 ± 0.01 ^b^	0.20 ± 0.01 ^ab^	0.15 ± 0.01 ^a^	0.21 ± 0.01 ^ab^	0.24 ± 0.04 ^b^	0.17 ± 0.02 ^a^	0.15 ± 0.01 ^a^
Ethyl palmitate	0.44 ± 0.04	0.44 ± 0.28	0.57 ± 0.08	0.49 ± 0.11	0.50 ± 0.04	0.44 ± 0.07	0.45 ± 0.08	0.46 ± 0.06	0.63 ± 0.09	0.61 ± 0.21	0.53 ± 0.24
Methyl stearate	n.d.	0.11 ± 0.05	0.2 ± 0.04	0.16 ± 0.05	0.16 ± 0.01	0.16 ± 0.02	0.1 ± 0.04	0.14 ± 0.03	0.10 ± 0.03	0.15 ± 0.05	0.13 ± 0.05
Ethyl linoleate	0.04 ± 0.00	0.09 ± 0.08	0.07 ± 0.01	0.04 ± 0.00	0.04 ± 0.01	0.07 ± 0.03	0.04 ± 0.01	0.07 ± 0.02	0.05 ± 0.00	0.06 ± 0.02	0.04 ± 0.02
Ethyl oleate	0.32 ± 0.02	0.48 ± 0.37	0.48 ± 0.05	0.31 ± 0.04	0.35 ± 0.05	0.47 ± 0.13	0.34 ± 0.07	0.45 ± 0.08	0.45 ± 0.03	0.49 ± 0.15	0.31 ± 0.13
Ethyl stearate	0.24 ± 0.03	0.27 ± 0.15	0.32 ± 0.01	0.30 ± 0.02	0.31 ± 0.05	0.29 ± 0.04	0.27 ± 0.03	0.29 ± 0.04	0.40 ± 0.03	0.39 ± 0.12	0.37 ± 0.17
∑Free fatty esters	1.49 ± 0.09	1.58 ± 0.88	1.8 ± 0.19	1.55 ± 0.15	1.61 ± 0.07	1.63 ± 0.28	1.35 ± 0.21	1.62 ± 0.22	1.86 ± 0.20	1.87 ± 0.56	1.53 ± 0.56

The value of volatile compounds is expressed as mean ± standard deviation (n = 3). Different letters in the column indicate a significant difference (*p* < 0.05). Abbreviations: 1,2-dioleoyl_GLP_Na—1,2-dioleoyl-sn-glycero-3-phospho-rac-(1-glycerol) sodium salt; 1-linoleoyl-rac-GL—1-linoleoyl-rac glycerol; *Sc- Saccharomyces cerevisiae.*

## Data Availability

The original data presented in the study are openly available in Dabar at https://urn.nsk.hr/urn:nbn:hr:293:080752 (accessed on 15 January 2024).
